# Microwave Breast Sensing via Deep Learning for Tumor Spatial Localization by Probability Maps

**DOI:** 10.3390/bioengineering10101153

**Published:** 2023-10-02

**Authors:** Marijn Borghouts, Michele Ambrosanio, Stefano Franceschini, Maria Maddalena Autorino, Vito Pascazio, Fabio Baselice

**Affiliations:** 1 Department of Biomedical Engineering, Technical University of Eindhoven, 5600 MB Eindhoven, The Netherlands; m.m.borghouts@student.tue.nl; 2 Department of Economics, Law, Cybersecurity and Sports Sciences, University of Naples “Parthenope”, 80035 Nola, Italy; 3 Department of Engineering, University of Napoli “Parthenope”, 80143 Naples, Italy; stefano.franceschini@uniparthenope.it (S.F.); mariamaddalena.autorino001@studenti.uniparthenope.it (M.M.A.); vito.pascazio@uniparthenope.it (V.P.); fabio.baselice@uniparthenope.it (F.B.)

**Keywords:** microwave imaging, neural networks, tumor localization, breast cancer, early detection, biomedical engineering

## Abstract

Background: microwave imaging (MWI) has emerged as a promising modality for breast cancer screening, offering cost-effective, rapid, safe and comfortable exams. However, the practical application of MWI for tumor detection and localization is hampered by its inherent low resolution and low detection capability. Methods: this study aims to generate an accurate tumor probability map directly from the scattering matrix. This direct conversion makes the probability map independent of specific image formation techniques and thus potentially complementary to any image formation technique. An approach based on a convolutional neural network (CNN) is used to convert the scattering matrix into a tumor probability map. The proposed deep learning model is trained using a large realistic numerical dataset of two-dimensional (2D) breast slices. The performance of the model is assessed through visual inspection and quantitative measures to assess the predictive quality at various levels of detail. Results: the results demonstrate a remarkably high accuracy (0.9995) in classifying profiles as healthy or diseased, and exhibit the model’s ability to accurately locate the core of a single tumor (within 0.9 cm for most cases). Conclusion: overall, this research demonstrates that an approach based on neural networks (NN) for direct conversion from scattering matrices to tumor probability maps holds promise in advancing state-of-the-art tumor detection algorithms in the MWI domain.

## 1. Introduction

Breast cancer, specifically the female variant, is the most prevalent type of cancer worldwide [1]. Statistics indicate that approximately one in eight new cancer cases falls under this category [2]. Moreover, breast cancer is the leading cause of mortality in women worldwide [1]. Fortunately, early detection of breast tumors through population screening has proven to be an effective strategy in reducing the societal impact and mortality rates associated with this disease [3].

Currently, medical imaging techniques such as X-ray mammography (gold standard), ultrasound (US) imaging, magnetic resonance imaging (MRI) and nuclear imaging are employed for breast cancer detection [4]. However, each of these modalities has its distinct drawbacks [4,5]. X-ray mammography and nuclear imaging involve the exposure of patients to harmful ionizing radiation. Furthermore, nuclear imaging and MRI are costly and time-consuming examinations. In addition, patients may experience discomfort during X-ray mammography due to required breast compression; nuclear imaging may cause discomfort due to the injection of radioactive material into the bloodstream; and MRI scanners can produce uncomfortable loud noises and require the patient to remain still in an enclosed space for extended periods of time. Lastly, US imaging is relatively operator-dependent, and correctly interpreting the images requires skilled clinicians. Given these challenges, microwave sensing can represent a valuable alternative to traditional medical exams and is a good candidate for large-scale early breast cancer screening because of its safety (non-ionizing radiation), cost-effectiveness, and time-efficient nature. Furthermore, this kind of examination is relatively comfortable and operator-independent [6].

To generate medical images from data collected by the microwave system, an inverse scattering problem has to be solved [7,8]. This problem entails reconstructing the morphological and/or electric properties of the scattering objects from the scattered waves, which are detected by microwave probing sensors. This kind of inverse problem is well-known in the scientific literature as the “inverse scattering problem” (ISP), and finding a stable solution is not trivial due to the issues of nonlinearity and ill-posedness [9]. Several inversion algorithms can be adopted to solve the ISP under consideration [10,11,12,13,14,15,16], and among them, neural networks seem to have better image reconstruction performance compared to traditional approaches [17,18,19,20,21,22].

Existing research in the field of microwave imaging has demonstrated the potential of artificial neural networks in reconstructing realistic breast images [20,21,23]. However, the reconstruction performance of these models in terms of both spatial resolution and retrieved complex permittivity values often remain insufficient to allow for accurate differentiation between breast tumors and the surrounding fibro-glandular tissue. Consequently, clinicians who rely on these images to form a diagnosis may struggle to do so with confidence.

Ideally, the considered imaging modality should support clinicians’ decision-making by providing a probability map, showing for each pixel/voxel the probability of it containing a tumor. Other researchers [24,25,26,27,28,29,30,31] have developed various approaches to provide similar information on the dimensions and location of the breast tumor in MWI. In [26,27,28,30] additional ultrasound data is incorporated to help with detection of tissue boundaries. In [26] for example, a multi-input multitask convolutional neural network (CNN) is provided that takes both electromagnetic (EM) data from MWI and ultrasound data, near the output the model splits into two separate processing paths to produce two outputs: a regression output and segmentation output of several tissue classes, including tumor class. In the study of [29] an, eigenfunction-based reconstruction of the complex permittivity is used as prior information for the contrast source inversion algorithm to improve the reconstructed images and subsequent tumor segmentation. Additionally, in [31] the Gauss-Newton inversion is used to form images of the complex permittivity and of the ultrasonic properties (compressibility and attenuation). These images are then processed using a CNN classifier which returns probability maps for five different tissue types including tumor. However, all these approaches use the inherently flawed reconstructed breast properties in some part of their approach for localizing potential tumors.

Conversely from the previously-mentioned articles, this study proposes a novel strategy in which a direct conversion is made from the raw microwave data into a spatial probability map of the tumors. This procedure allows to skip the image formation step, which is particularly difficult and, sometimes, unstable, leading to potential information loss and ambiguity about the tumor localization. To the best of our knowledge, only [32,33] have published approaches for such a direct conversion from scattered wave data into a tumor location in the microwave imaging domain. However, the proposed framework in [32] only estimates the quadrant of the image in which the tumor resides. In [33], their framework only estimates the spatial coordinates of the tumor center. Furthermore, the profiles in their dataset are limited to having a single smooth tumor of fixed size. Since the tumor probability maps in our approach are derived directly from the scattering matrices, they remain independent of any specific image formation strategy. Consequently, the tumor probabilities generated by this approach can be synergistically combined with various image formation approaches that also take the scattering matrices as input. In such cases, the tumor probability map can provide accurate information regarding tumor size and location, while the accompanying retrieved image supplies contextual information relevant to the tumor position.

This study aims to build on the work of [20,34] by developing a neural network model that generates a pixel-wise tumor probability map directly from the scattering matrix. The performance of this model will be evaluated through various quantitative and visual assessments. The ultimate objective of this research is to advance the current state-of-the-art methodology for breast cancer screening, thereby mitigating the adverse impact of the disease.

Section 2 of this paper will summarize the fundamental aspects of microwave sensing and imaging. Section 3 will provide detailed explanations of the employed methodology, including the dataset characteristics, the neural network architecture, the training process, and the performance evaluation. In Section 4, the results will be presented, consisting of both numerical and visual aspects. The final sections will involve the interpretation of the results (Section 5), drawing the conclusions (Section 6), and reflecting upon the methodology as a whole.

## 2. Microwave Sensing

**Figure 1:**
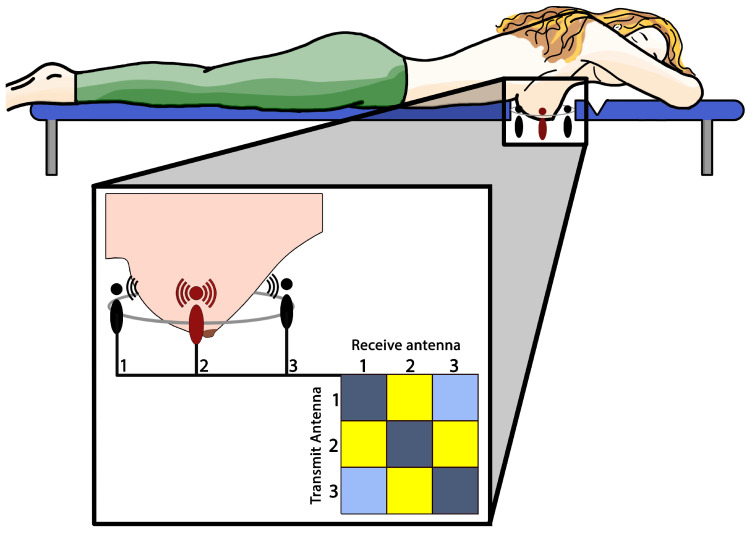
Simplified sketch of a microwave breast imaging setup with three antennas. The red antenna (2) transmits an electromagnetic wave, the other antennas (1,3) receive the scattered waves. This procedure is repeated per each antenna and the collected data are stored in the form of the scattering matrix.

Microwave sensing relies on the use of electromagnetic waves within the microwave frequency range, spanning from several hundreds of MHz to multiple GHz [6,35,36]. By processing the waves scattered by breast tissues, internal structural images of the breast can be constructed. Typically, an arrangement of antennas encircling the breast is employed for imaging purposes. These antennas surround the imaging domain and can be mounted along the edge of a spherical indentation in a medical exam table. Such a setup is particularly comfortable since it does not require breast compression (Figure 1).

During signal acquisition, the system operates in a multi-view-multi-static configuration, referring to the fact that more transmitters and receivers, located in different spatial locations, compose the system. More in detail, only one antenna at a time works as transmitter, emitting a microwave signal that propagates through the breast tissues and, by virtue of the contrast in the electric properties existing between adjacent biological tissues, the scattered waves can then be detected by the receiving antennas outside the breast.

At the end of the acquisition phase, a collection of scattered signals is obtained resulting from each pair of transmitting-receiving antennas. This information can be properly re-arranged in a matrix form whose size coincides with the number of transmitting and receiving elements. It is worth noting that the considered system collects complex information, which can also be adopted to perform a “coherent” imaging of the area under investigation. Generally, complementary to the spatial variability information which is exploited by changing the relative position between transmitter and receiver, further important information for improving the quality of the investigation and empowering the diagnosis consists in the adopted frequency range, allowing improved resolution in the tumor detection/localization performance.

Nevertheless, there are some lower bounds in the resolution capabilities of a well-designed microwave system, since the choice of the adopted frequency range is the result of the trade-off between penetration capability and spatial resolution performance [23]. Furthermore, it is paramount to underline that an increase in the number of transmitting/receiving antennas does not imply an improvement in terms of detection/localization performance necessarily, as supported by the well-known theory on the degrees of freedom [37,38], but still such an improvement can reduce the impact of noise on the detection/imaging tasks.

Further details on the mathematical equations ruling the scattering phenomena, which is out of the scope of this work, can be found in [39]. Nevertheless, it is worth underlining that due to the complexity of the problem under consideration, performing the imaging to carry out the tumor diagnosis represents a hard task which is still very challenging for several state-of-the-art microwave imaging approaches, even for the most recent ones based on the use of NNs, due to the main issues related to the non-linearity and ill-posedness of the inverse scattering problem [9]. In the light of this, the adoption of NNs for a different task, such as the one of directly estimating tumor location without performing the imaging of the breast, can represent a promising strategy.

## 3. Methodology

### 3.1. Neural Network Design

Probability maps regarding the tumors dimensions and location would be a valuable tool in breast cancer screening. In this framework, the proposed approach involves a neural network model that creates such a probability map directly from the scattering matrix. The developed framework in this study consists of a U-Net architecture followed by a single dense layer. It is worth noting that even though further architectures might represent better options, such as the ones in [40,41,42], we chose the U-net as it represents a good trade-off among performance, complexity of the architecture and training time.

**Figure 2:**
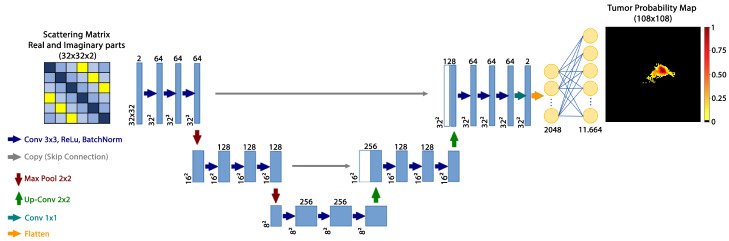
The proposed neural network architecture. The model takes as input the complex scattering matrix re-arranged into real and imaginary parts. The model outputs a pixel-wise tumor probability map.

The proposed architecture operates on the scattering matrix and is three levels deep; each convolutional block uses three 3×3 convolutions. Every convolution is followed by a ReLu activation and a batch normalization. In these blocks, padded convolutions are performed which causes the model to maintain the input image dimensions at the output. Subsequently, the resulting output is flattened and passed to a fully connected dense layer to enable the transformation of the data into the desired output shape. Figure 2 shows this architecture.

### 3.2. Dataset Characteristics

An adequately large and diverse dataset is essential to effectively train neural networks. However, in the domain of microwave imaging, obtaining such datasets is challenging due to the limited implementation of this technology on a large scale in clinical settings. Secondly, clinical data usually lacks the reference image that is needed to train a neural network. To address these issues, ref. [20] developed a realistic numerical two-dimensional breast phantom generator, which has been made available for this study.

**Figure 3:**
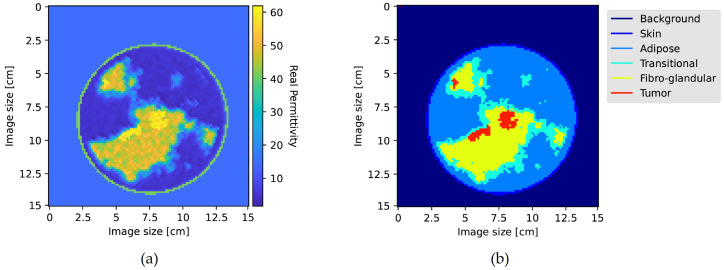
(**a**) the real permittivity of a generated profile. (**b**) the segmented version of the same profile.

Following the generation of 2D phantoms, a microwave imaging dataset was created in accordance with the methodology outlined in [20]. In this process, breast slices measuring 15×15 square centimeters were generated. The images were saved with a resolution of 7.2 pixels per centimeter, equating to 108×108 pixels per each breast image. The breast slices were subsequently categorized into one of four classes as defined in [20], based on the relative percentage of fibro-glandular tissue with respect to the other tissues. Each pixel of the generated breast profiles was classified into six distinct tissue classes. Subsequently, the tumor class was extracted to create binary tumor labels, which serve as the ground truth for training the model. Figure 3 provides a sketch, showcasing the real permittivity of a generated profile of a tumorous breast as well as the segmented image.

Similar to the procedure in [20], the imaging setup was assumed to have 30 antennas, thus the corresponding scattering matrix has size 30×30. The scattering matrices are calculated based on a forward solver which exploits the method of moments (MoM) to solve the forward scattering problem. It is worth noting that the scattering matrix is complex; consequently, for each breast slice, two 30×30 matrices were adopted, one containing the real values and the other containing the imaginary values. As a final step, these matrices were padded with a single layer of zeros, extending their size to 32×32. This step was necessary to ensure compatibility with the U-net architecture.

### 3.3. Network Training

For the training and evaluation of the model, a total of 160,000 profiles were generated, evenly distributed among the four predefined classes. The breast profiles were combined in a 1:1 ratio of healthy and tumorous profiles. Upon request, this data can be made available for research purposes. Of these profiles, 128,000 were allocated for training, 16,000 for validation, and an additional 16,000 for testing purposes, following an 80–10–10% split. During training, the model attempted to minimize the binary cross-entropy loss (BCELoss). Optimization was performed using the Adam algorithm, employing an initial learning rate of 0.00123. The training process was conducted in mini-batches of 500 profiles. Training was performed on an NVIDIA Quadro RTX 6000 GPU, with each epoch taking approximately 50 s using the specified settings. A minimal validation BCELoss of 0.01595 was achieved after 19 epochs before the model started over-fitting.

### 3.4. Performance Assessment

To assess the quality of the estimated tumor probability maps, various metrics were applied to the independent test set. These metrics were chosen to assess the model’s performance at different levels.

The first metric aims to measure model performance at the sample level, specifically its ability to differentiate between healthy and diseased samples by classifying them into their respective categories. This type of performance is particularly valuable for efficient breast cancer screening, where samples that are labeled as diseased can be examined further using other modalities with superior image quality.

The second metric focused on assessing the model’s performance at a regional level, specifically its capability to locate the general area of a single tumor. This was achieved by calculating the distance between the real tumor center and the estimated tumor center. This type of performance provides clinicians with valuable initial information about the tumor’s approximate location within the breast.

The third metric aims to evaluate the model’s performance at a highly detailed level by assessing the accuracy of the probability maps pixel-by- pixel. Three image similarity metrics were employed for this purpose. This level of performance is crucial for precise treatment planning and monitoring subtle changes of the tumor over time.

For the first metric (global classification), the tumor probabilities estimated by the network were transformed into binary labels using a threshold of 15%. If any pixel within a breast profile exceeded this threshold, the profile was classified as containing a tumor. Based on these binary labels, the model’s classification accuracy, specificity, and sensitivity were calculated.

For the second metric (regional locating), a subset of the independent test dataset was selected, consisting of profiles that contained a single connected tumor while maintaining an equal distribution across the four predefined classes of [20]. The centers were determined with the formula for the center of mass, swapping mass for tumor probability. This calculation was followed by discretisation to the nearest integer to obtain the index of the central pixel. Finally, the Euclidean distance between the real and estimated centers was computed.

For the third metric (detailed pixel-wise), the soft-Dice score [43], the normalized cross correlation (NCC) [44], and the normalized root mean square error (NRMSE) [45] were employed to measure the overlap between the estimated tumor map and the reference. All three of these image similarity metrics (Equations (1)–(3)) are able to accommodate the disparity between the outputted image, which is a probability map ranging from 0 to 1, and the reference, which is a binary map consisting exclusively of 0 s and 1 s.

(1) $$\mathrm{Soft-Dice}\left(I_{1},I_{2}\right)=\frac{2\sum_{x,y}I_{1}\left(x,y\right)\cdot I_{2}\left(x,y\right)}{\sum_{x,y}I_{1}^{2}\left(x,y\right)+\sum_{x,y}I_{2}^{2}\left(x,y\right)}$$

(2) $$\mathrm{NCC}\left(I_{1},I_{2}\right)=\frac{\sum_{x,y}\left(I_{1}\left(x,y\right)-\mu_{I_{1}}\right)\left(I_{2}\left(x,y\right)-\mu_{I_{2}}\right)}{\sqrt{\sum_{x,y}{\left(I_{1}\left(x,y\right)-\mu_{I_{1}}\right)}^{2}\sum_{x,y}{\left(I_{2}\left(x,y\right)-\mu_{I_{2}}\right)}^{2}}}$$

(3) $$\mathrm{NRMSE}\left(I_{1},I_{2}\right)=\frac{\sqrt{\frac{1}{N}\sum_{x,y}{\left(I_{1}\left(x,y\right)-I_{2}\left(x,y\right)\right)}^{2}}}{\max\left(I_{1}\right)-\min\left(I_{1}\right)}$$

In Equations (1)–(3), $I_{1}$ and $I_{2}$ represent the two input images, x and y represent the spatial coordinates of the images, $\sum_{x,y}$ represents a summation over all the pixels, *N* denotes the total number of pixels in each image, and $\mu_{I_{1}}$ and $\mu_{I_{2}}$ denote the mean intensities of $I_{1}$ and $I_{2}$, respectively. Note that in the Equation (3), image 1 is assumed to have the largest intensity range. In the case of binary masks, the data range equals 1 so the RMSE is normalized by default.

## 4. Results

### 4.1. Visual Analysis

**Figure 4:**
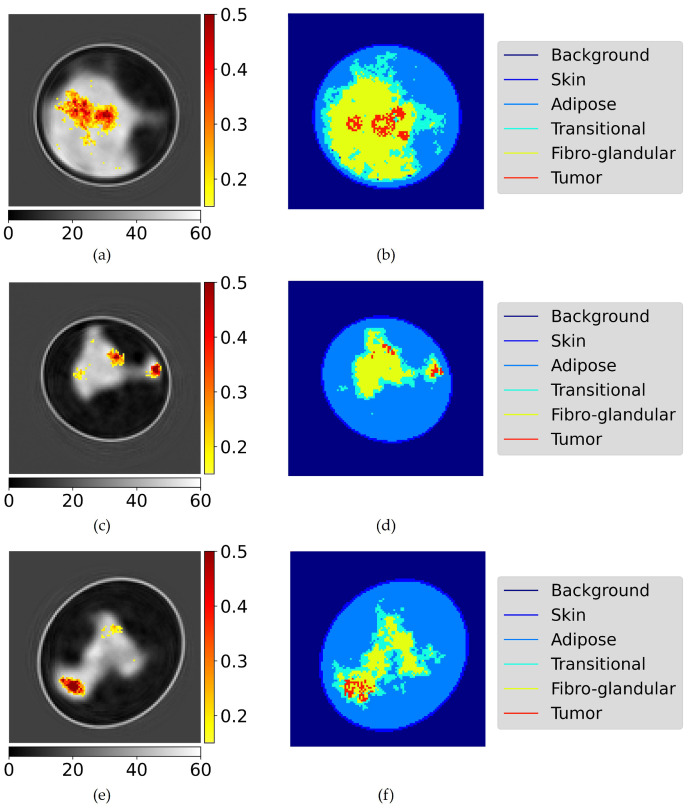
(**a**,**c**,**e**) estimated probability maps with the corresponding generated breast image as background. (**b**,**d**,**f**) corresponding segmented reference profiles. (**a**,**b**) example of a very dense breast which is notoriously difficult for tumour detection. (**c**,**d**) example of a breast with small and scattered tumours. (**e**,**f**) example of a breast with a single connected tumour. The images are of size 15×15 cm^2^. The vertical colorbar represents the tumour probability, the horizontal colorbar represents the real permittivity.

The proposed model in this research generates a tumor probability map. Although this probability map possesses intrinsic value, its interpretability and informativeness are enhanced when it is combined with a breast image generated by a state-of-the-art image formation model. In the presented results, a neural network for image formation was used following the approach stated in [20], since they have shown good results. Figure 4 shows three generated probability maps, combined with the generated images in grayscale. Adjacent to these estimations are the segmented reference images.

### 4.2. Classification

The primary objective of the classification metrics is to assess the effectiveness of the model in its fundamental screening task, which involves discerning between healthy subjects and subjects with a tumor. The provided information in Table 1 illustrates the confusion matrix for classification of breast profiles, categorizing them as either “healthy” (negative) or “malignant” (positive). The testing dataset consists of a distribution of approximately 50% (8004) healthy profiles and 50% (7996) malignant profiles. Consequently, Table 2 shows the corresponding accuracy, sensitivity, specificity, F1 score and precision. Furthermore, the ROC-AUC (receiver operating characteristic-area under the curve) was evaluated and it is equal to 0.9992.

### 4.3. Distance between Tumor Centers

**Figure 5:**
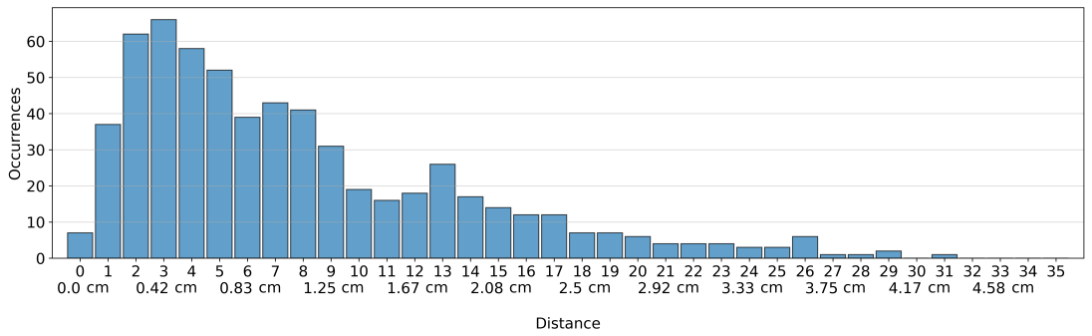
Distribution of Euclidean distances between the estimated center and the actual center of the tumor. The histogram contains 620 occurrences. The distance is given in pixels for every bin and in centimeters for every third bin. One pixel = 0.139 cm.

The objective of this metric is to measure the model’s ability to pinpoint the center of a single tumor. Figure 5 presents a histogram that depicts the distribution of Euclidean distances between the estimated center and the actual center of the tumor. From Figure 5, it can be observed that the model distance is approximately 0.4 cm (3 pixels). The average distance measures around 1.2 cm (8.64 pixels) with a standard deviation of 0.9 cm (6.49 pixels). Additionally, more than 50% of samples have an error of less than 0.9 cm (6.71 pixels).

### 4.4. Pixel-Wise Image Similarity

This final metrics measure the similarity between the probability map and the reference map from pixel-to-pixel. For this analysis, the soft-Dice, the normalized cross correlation, and the normalized root mean square error were calculated. The previous results in Table 2 show the remarkably high classification accuracy that was achieved, with only eight errors observed among the 16,000 testing profiles. Given this exceptional accuracy, it was decided to exclusively apply the predefined image similarity metrics to the malignant profiles. This approach ensures that the metrics remain unaffected by the 50% contribution from the nearly perfect probability maps associated with the non-tumorous breast profiles. Additionally, the calculation of the Normalized Root Mean Squared Error (NRMSE) was confined to the pixels identified as tumors in the reference image or estimated as such. This restriction aims to prevent the metric from being heavily influenced by the many accurately classified background pixels resulting from the class imbalance between background and tumor class. The specific metric values are presented in Table 3.

## 5. Discussion

This research paper proposed a novel deep learning approach aimed at reconstructing a spatial tumor probability map in the domain of microwave imaging. The proposed approach pioneers the use of the scattering matrix to obtain a probability map of the spatial tumor localization, circumventing the use of inherently flawed reconstructed images which many other approaches rely on for tumor detection and localization. Improvements in this field are crucial to advance population screening techniques for breast tumors and mitigate the disease impact at both individual and societal level.

The developed model utilizes a U-Net architecture and a fully connected layer. To train and evaluate the model, a large and diverse synthetic dataset was employed. This dataset was generated using the data generator developed by [20]. The resulting model was subjected to various performance measures aimed to evaluate the performance of the model at different resolutions.

The obtained results, as depicted in Table 1 and Table 2, demonstrate the remarkable accuracy of this approach in distinguishing between healthy and malignant profiles, even when dealing with tumors as small as a few millimeters in size. Furthermore, the dataset encompasses cases of dense breasts, known for their challenges in tumor identification. The proposed approach outperforms a similar NN approach for sample classification described in [34], which uses the same data generator. This approach reached a classification accuracy of 0.995 compared to 0.9995 presented in this paper. This high classification accuracy holds great value for large-scale population screening, given the fast, comfortable, safe, and cost-effective nature of microwaves as diagnostic tool. Individuals identified as diseased through this approach can subsequently undergo scanning with other modalities that offer superior image quality, aiding in further treatment planning.

The results in Figure 5 illustrate the ability of our approach to accurately locate the center of a single tumor, with an error margin of less than 0.9 cm achieved in over 50% of cases. When combined with the reconstructed image of the breast, this information can provide clinicians with an initial understanding of the tumor’s relative location within the breast.

Lastly, the image similarity metrics employed to assess the overlap between the real and estimated tumors, as shown in Table 3, yield relatively low scores. This is partially due to the modest confidence of the model, seldom exceeding 0.4. Consequently, even a geometrically precise prediction with this level of confidence will result in overlap scores below 0.4. However, considering this partial explanation it may still be concluded that the model is not able to accurately determine the exact dimensions and size of tumor, as evidenced by the visual results. Though this outcome was anticipated due to the subtle variation in electrical permittivity between tumor tissue and its surrounding tissue, as well as the presence of a gradient rather than a distinct boundary between these tissues. Moreover, the intrinsic complexity of the problem under consideration contributes to the limited quality of the generated tumor probability maps, as evidenced by the reconstruction quality of state-of-the-art image formation techniques.

## 6. Conclusions

In conclusion, the proposed strategy demonstrates superior classification performance in comparison to the current state-of-the-art methods, such as [34]. Furthermore, the proposed model demonstrates an ability to accurately identify the center of an individual tumor with a margin of error within 0.9 cm for the majority of cases. These advancements contribute positively to improving the state-of-the-art method for breast cancer screening, which helps reduce the impact of the disease.

It is worth noticing that the proposed approach was designed for a pre-screening phase, i.e., for early breast cancer diagnosis, being characterized by an excellent pre-screening power and providing very good initial information. Thus, the main idea is that this cutting-edge technology can work in synergy with more traditional and better-performing imaging technologies in a multi-step fashion, in which the preliminary screening and localization can be performed by means of microwaves, just improving the safety for the patient and reducing the costs for the health care system, while referring to conventional medical imaging technologies for further investigations and clinical decisions.

Future research will focus on expanding the existing approach to encompass three-dimensional reconstructions and the testing on more realistic scenarios. Additionally, the probability maps can be up-scaled or down-scaled to see how this change in resolution affects the model’s performance.

## Figures and Tables

**Table 1:** Confusion matrix for breast profile classification (positive = malignant, negative = healthy).

		Predicted	
		**Positive**	**Negative**
**Actual**	**Positive**	7993	3
	**Negative**	5	7999

**Table 2:** Metrics for breast profile classification performance.

Metric	Formula	Value
Accuracy	$\frac{TP+TN}{TP+TN+FP+FN}$	0.9995
Sensitivity (Recall)	$\frac{TP}{TP+FN}$	0.9996
Specificity	$\frac{TN}{TN+FP}$	0.9994
Precision	$\frac{TP}{TP+FP}$	0.9994
F1 Score	$\frac{2TP}{2TP+FP+FN}$	0.9995

**Table 3:** Pixel-based image similarity metrics.

Metric	Mean	Standard Deviation
Soft-Dice	0.144	0.078
NCC	0.337	0.148
NRMSE	0.809	0.057

## Data Availability

Not applicable.

## Abbreviations

The following abbreviations are used in this manuscript:

MWI	microwave imaging
CNN(s)	convolutional neural network(s)
2D	two-dimensional
NN(s)	neural network(s)
US	ultrasound
MRI	magnetic resonance imaging
ISP	inverse scattering problem
EM	electromagnetic
MoM	method of moments
BCELoss	binary cross-entropy loss
NCC	normalised cross-correlation
NRMSE	normalised root mean square error

## References

[1] Arnold M., Morgan E., Rumgay H., Mafra A., Singh D., Laversanne M., Vignat J., Gralow J., Cardoso F., Siesling S. (2022). Current and future burden of breast cancer: Global statistics for 2020 and 2040. Breast.

[2] Sung H., Ferlay J., Siegel R., Laversanne M., Soerjomataram I., Jemal A., Bray F. (2021). Global Cancer Statistics 2020: GLOBOCAN Estimates of Incidence and Mortality Worldwide for 36 Cancers in 185 Countries. CA Cancer J. Clin..

[3] Boyle P., IARC (2008). World Cancer report 2008. Cancer Control.

[4] Jaglan P., Dass R., Duhan M. (2019). Breast Cancer Detection Techniques: Issues and Challenges. J. Inst. Eng. Ser. B.

[5] Katz R., Wilson L., Frazer N. (1994). Anxiety and its determinants in patients undergoing Magnetic Resonance Imaging. J. Behav. Ther. Exp. Psychiatry.

[6] Nikolova N. (2011). Microwave imaging for breast cancer. IEEE Microw. Mag..

[7] Hopcraft K., Smith P. (2013). An Introduction to Electromagnetic Inverse Scattering.

[8] Fear E., Meaney P., Stuchly M. (2003). Microwaves for breast cancer detection?. IEEE Potentials.

[9] Colton D., Kress R. (2019). Inverse Acoustic and Electromagnetic Scattering Theory.

[10] Bevacqua M., Di Meo S., Crocco L., Isernia T., Pasian M. (2021). Millimeter-waves breast cancer imaging via inverse scattering techniques. IEEE J. Electromagn. Microwaves Med. Biol..

[11] Scapaticci R., Kosmas P., Crocco L. (2014). Wavelet-based regularization for robust microwave imaging in medical applications. IEEE Trans. Biomed. Eng..

[12] Reimer T., Solis-Nepote M., Pistorius S. (2020). The application of an iterative structure to the delay-and-sum and the delay-multiply-and-sum beamformers in breast microwave imaging. Diagnostics.

[13] Poli L., Oliveri G., Massa A. (2012). Microwave imaging within the first-order Born approximation by means of the contrast-field Bayesian compressive sensing. IEEE Trans. Antennas Propag..

[14] Cuccaro A., Dell’Aversano A., Ruvio G., Browne J., Solimene R. (2021). Incoherent radar imaging for breast cancer detection and experimental validation against 3D multimodal breast phantoms. J. Imaging.

[15] Berg P., Kleinman R. (1997). A contrast source inversion method in the wavelet domain. Inverse Probl..

[16] Cui T., Chew W., Aydiner A., Chen S. (2001). Inverse scattering of two-dimensional dielectric objects buried in a lossy earth using the distorted born iterative method. IEEE Trans. Geosci. Remote Sens..

[17] Chen X., Wei Z., Maokun L., Rocca P. (2020). A review of deep learning approaches for inverse scattering problems (invited review). Electromagn. Waves.

[18] Dachena C., Fedeli A., Fanti A., Lodi M., Fumera G., Pastorino M., Razzo A. (2022). Initial experimental tests of an ANN-based microwave imaging technique for neck diagnostics. IEEE Microw. Wirel. Components Lett..

[19] Li X., Zhou Y., Wang J., Wang Q., Lu Y., Duan X., Sun Y., Zhang J., Liu Z. (2019). A novel deep neural network method for electrical impedance tomography. Trans. Inst. Meas. Control.

[20] Ambrosanio M., Franceschini S., Pascazio V., Baselice F. (2022). An End-to-End Deep Learning Approach for Quantitative Microwave Breast Imaging in Real-Time Applications. Bioengineering.

[21] Wang L. (2022). Holographic Microwave Image Classification Using a Convolutional Neural Network. Micromachines.

[22] Shao W., Du Y. (2020). Microwave Imaging by Deep Learning Network: Feasibility and Training Method. IEEE Trans. Antennas Propag..

[23] AlSawaftah N., El-Abed S., Dhou S., Zakaria A. (2022). Microwave imaging for early breast cancer detection: Current state, challenges, and future directions. J. Imaging.

[24] Edwards K., Khoshdel V., Asefi M., Lovetri J., Gilmore C., Jeffrey I. (2021). A machine learning workflow for tumour detection in breasts using 3d microwave imaging. Electronics.

[25] Dey M., Rana S., Loretoni R., Duranti M., Sani L., Vispa A., Raspa G., Ghavami M., Dudley S., Tiberi G. (2022). Automated breast lesion localisation in microwave imaging employing simplified pulse coupled neural network. PLoS ONE.

[26] Qin Y., Ran P., Rodet T., Lesselier D. (2022). Breast imaging by convolutional neural networks from joint microwave and ultrasonic data. IEEE Trans. Antennas Propag..

[27] Omer M., Mojabi P., Abdollahi N., Kurrant D., Jeffrey I., LoVetri J., Fear E. Breast Imaging with Multiphysics Prior for Improved Tumour Detection and Localization. Proceedings of the ANTEM 2018: 2018 18th International Symposium on Antenna Technology and Applied Electromagnetics.

[28] Qin Y., Rodet T., Lesselier D., Member S. (2022). Fused Microwave and Ultrasonic Breast Imaging Within the Framework of a Joint Variational Bayesian Approximation. IEEE Trans. Antennas Propag..

[29] Abdollahi N., Jeffrey I., LoVetri J. (2020). Improved Tumor Detection via Quantitative Microwave Breast Imaging Using Eigenfunction-based Prior. IEEE Trans. Comput. Imaging.

[30] Qin Y., Rodet T., Lambert M., Lesselier D. (2020). Microwave Breast Imaging with Prior Ultrasound Information. IEEE Open J. Antennas Propag..

[31] Mojabi P., Khoshdel V., Lovetri J. (2020). Tissue-type classification with uncertainty quantification of microwave and ultrasound breast imaging: A deep learning approach. IEEE Access.

[32] Lu M., Xiao X., Pang Y., Liu G., Lu H. (2022). Detection and Localization of Breast Cancer Using UWB Microwave Technology and CNN-LSTM Framework. IEEE Trans. Microw. Theory Tech..

[33] Lu M., Xiao X., Liu G., Lu H. (2021). Microwave breast tumor localization using wavelet feature extraction and genetic algorithm-neural network. Med. Phys..

[34] Franceschini S., Autorino M., Ambrosanio M., Pascazio V., Baselice F. (2023). A Deep Learning Approach for Diagnosis Support in Breast Cancer Microwave Tomography. Diagnostics.

[35] Chandra R., Zhou H., Balasingham I., Narayanan R. (2015). On the opportunities and challenges in microwave medical sensing and imaging. IEEE Trans. Biomed. Eng..

[36] Wang L. (2023). Microwave Imaging and Sensing Techniques for Breast Cancer Detection. Micromachines.

[37] Bucci O., Franceschetti G. (1989). On the degrees of freedom of scattered fields. IEEE Trans. Antennas Propag..

[38] Bucci O., Franceschetti G. (1987). On the spatial bandwidth of scattered fields. IEEE Trans. Antennas Propag..

[39] Pastorino M. (2010). Microwave Imaging.

[40] Cao H., Wang Y., Chen J., Jiang D., Zhang X., Tian Q., Wang M. Swin-unet: Unet-like pure transformer for medical image segmentation. Proceedings of the European Conference on Computer Vision.

[41] Shen J., Lu S., Qu R., Zhao H., Zhang L., Chang A., Zhang Y., Fu W., Zhang Z. (2023). A boundary-guided transformer for measuring distance from rectal tumor to anal verge on magnetic resonance images. Patterns.

[42] Chen J., Lu Y., Yu Q., Luo X., Adeli E., Wang Y., Lu L., Yuille A., Zhou Y. (2021). Transunet: Transformers make strong encoders for medical image segmentation. arXiv.

[43] Müller D., Kramer F. (2021). MIScnn: A framework for medical image segmentation with convolutional neural networks and deep learning. BMC Med. Imaging.

[44] Eskicioglu A., Fisher P. (1995). Image Quality Measures and Their Performance. IEEE Trans. Commun..

[45] Sutton B., Noll D., Fessler J. (2003). Fast, iterative image reconstruction for MRI in the presence of field inhomogeneities. IEEE Trans. Med. Imaging.

